# Effect of an equipment-behavior change intervention on handwashing behavior among primary school children in Kenya: the Povu Poa school pilot study

**DOI:** 10.1186/s12889-019-6902-2

**Published:** 2019-05-28

**Authors:** Wit Wichaidit, Rachel Steinacher, Jemima Akinyi Okal, Jaynie Whinnery, Clair Null, Katarzyna Kordas, Jihnhee Yu, Amy J. Pickering, Pavani K. Ram

**Affiliations:** 1 Department of Epidemiology and Environmental Health, State University of New York at Buffalo, Buffalo, USA; 2 Innovations for Poverty Action, New Haven, USA; 3 Department of Biostatistics, State University of New York at Buffalo, Buffalo, USA; 4 Department of Civil and Environmental Engineering, Tufts University, Medford, USA

**Keywords:** Soapy water, Behavioral intervention, Hand hygiene, Compliance, Implementation research

## Abstract

**Background:**

Handwashing prevalence in schools in Kenya is low due to lack of access to water and soap and lack of drive for handwashing. Soapy water made from detergent powder is an inexpensive alternative to bar soap and disgust and social norms change can be powerful drivers of handwashing, but their effectiveness has not been assessed in school setting. In Kenyan public schools, we evaluated an equipment-behavior change intervention’s effect on handwashing outcomes. We also monitored functionality of the Povu Poa prototypes to identify design improvements necessary for continued high usage in institutional settings.

**Methods:**

The intervention included the “Povu Poa”, a new type of handwashing station that dispensed foaming soap and rinse water, combined with school-wide behavior change promotion based on disgust and social norms. In this stepped-wedge cluster-randomized trial, we randomly selected 30 schools and divided them into 3 groups of 10. Following baseline data collection, we delivered the intervention sequentially (Group 1: 3–5 weeks after baseline; Group 2: 6–8 weeks; Group 3: 19–24 weeks). We observed outcomes [1] availability of handwashing materials at handwashing places, and; 2) observed handwashing behavior after toilet use among schoolchildren) at baseline and in three follow-up rounds. We compared the outcomes between schools that had received the intervention and schools that had not yet received the intervention.

**Results:**

Water and soap/soapy water were available at 2% of school visits before intervention, and at 42% of school visits after intervention.. Before intervention, we observed handwashing with water after 11% of 461 toilet use events; no one was observed to wash hands with soap/soapy water. After intervention, we observed handwashing after 62% of 383 toilet use events (PR = 5.96, 95% CI = 3.02, 11.76) and handwashing with soap/soapy water after 26% of events (PR incalculable). Foaming soap dispenser caps were cracked in 31% of all observations, but were typically still functional.

**Conclusions:**

Our combined equipment-behavior intervention increased availability of handwashing materials and improved the compliance with handwashing after using the toilet, but handwashing with soap was still rare. Equipment durability must be improved for deployment in schools at scale.

American Economic Association’s Registry for Randomized Controlled Trials; Trial Registry Number (TRN): AEARCTR-0000662; Date of Registry: April 14, 2015.

**Electronic supplementary material:**

The online version of this article (10.1186/s12889-019-6902-2) contains supplementary material, which is available to authorized users.

## Background

Diarrhea and respiratory infections are among the most common causes of death in children in low-income countries [1, 2]. Hand hygiene is a low-cost yet effective measure in preventing illness [3, 4]. Among schoolchildren, prevention of illnesses is important because of the direct health benefit of preventing diarrhea and respiratory infections [5, 6] and the indirect benefit of reducing absenteeism, both of which can affect educational attainment [7, 8].

Approximately 15% of the population in low and middle-income countries wash their hands with water and soap after fecal contact [9]. In school settings, there is low level of access to soap, and very low prevalence of handwashing. In Kenya, one cross-sectional study showed that only 14 to 18% of students had access to handwashing soap [10]. Evaluation of the SOPO handwashing promotion campaign in Kenyan primary schools showed that less than 2% of toilet use events were followed by handwashing with water and soap, 2 years after the intervention [11].

Previous interventions to increase handwashing in schools include efforts to overcome the lack of soap by budgeting for soap purchases [10, 12], hiring a WASH attendant to ensure presence of water and soap at handwashing place [12], using alcohol-based hand sanitizer as an alternative to bar soap [13, 14], and implementing a classroom-based multi-faceted intervention [15]. However, some of these interventions did not directly measure handwashing behavior as an outcome [15], and not all of them helped to prevent or mitigate loss or theft of bar soap. Furthermore, the cost of some of the interventions was high, rendering them impractical to scaling up in low-income countries.

Schools in Kenya often lack funds for purchasing soap, and bar soap tends to be stolen or lost [16], thus many students do not have soap to wash their hands. Soapy water, a mixture of powder detergent or liquid soap and water, has been shown to be microbiologically similar to bar soap for removal of microorganisms from hands [17]. In communities in western Kenya, the cost of soap per 100 handwashing events was less than $0.10 for soapy water compared to $0.20 to $0.44 for conventional bar soap [18]. The use of soapy water also deters soap theft and thus has the potential to help overcome lack of access to soap in schools [19]. In water-limited settings, the use of a foam dispenser also can help to further conserve soapy water and reduce the amount of water needed for rinsing. Assessment of the acceptability and effectiveness of foaming soap in schools would help to inform future handwashing promotion efforts for schools in resource-limited settings.

There is a well-recognized gap between knowledge about handwashing and actual handwashing practice, even following exposure to handwashing promotion [20–22]. Prior interventions have focused on health education rather than stronger behavioral determinants, such as emotional drivers [23] and social norms [10]. In a community setting in India, investigators found that behavioral change intervention using disgust-based triggers and nurture was associated with higher probability of handwashing with soap among mothers of schoolchildren (37% in the intervention group vs. 4% in the control group) [24]. A behavioral intervention using disgust as a behavioral driver has not been previously tested in school-age children, but would help inform future handwashing promotion efforts in school setting.

This study assessed the effect of providing Povu Poa handwashing stations with foaming soap dispensers combined with behavior promotion utilizing disgust and social norms in public primary schools in Western Kenya on two primary outcomes: 1) availability of handwashing materials at handwashing places; 2) observed handwashing behavior after toilet use among schoolchildren. We also used the school pilot as an opportunity to monitor the durability of the product equipment over a 1-year period in a high-usage setting to identify design improvements that would increase robustness and extend the product lifespan in school settings.

## Methods

### Povu Poa handwashing stations - school pilot study design

We conducted a cluster randomized trial using a stepped wedge design to evaluate the use of *Povu Poa* (“Cool Foam”) handwashing stations combined with a behavior intervention designed to change social norms and use disgust as a behavioral trigger. The Povu Poa handwashing stations are portable handwashing systems consisting of a water container in the form of a bucket or pipe, a wooden or plastic stand, a soap-frugal foaming dispenser (that converts soapy water into foam), and a water-conserving tap [18].

We chose the stepped-wedge design for several reasons. First, as the intervention posed no harm and brought benefit to the participating schools by creating places to wash hands with soap, it would be unethical to withhold the intervention in some participating schools. Second, the sequential roll-out of the intervention enabled the enumerators to deliver the intervention to the 30 participating schools while minimizing logistical constraints.

**Fig. 1:**
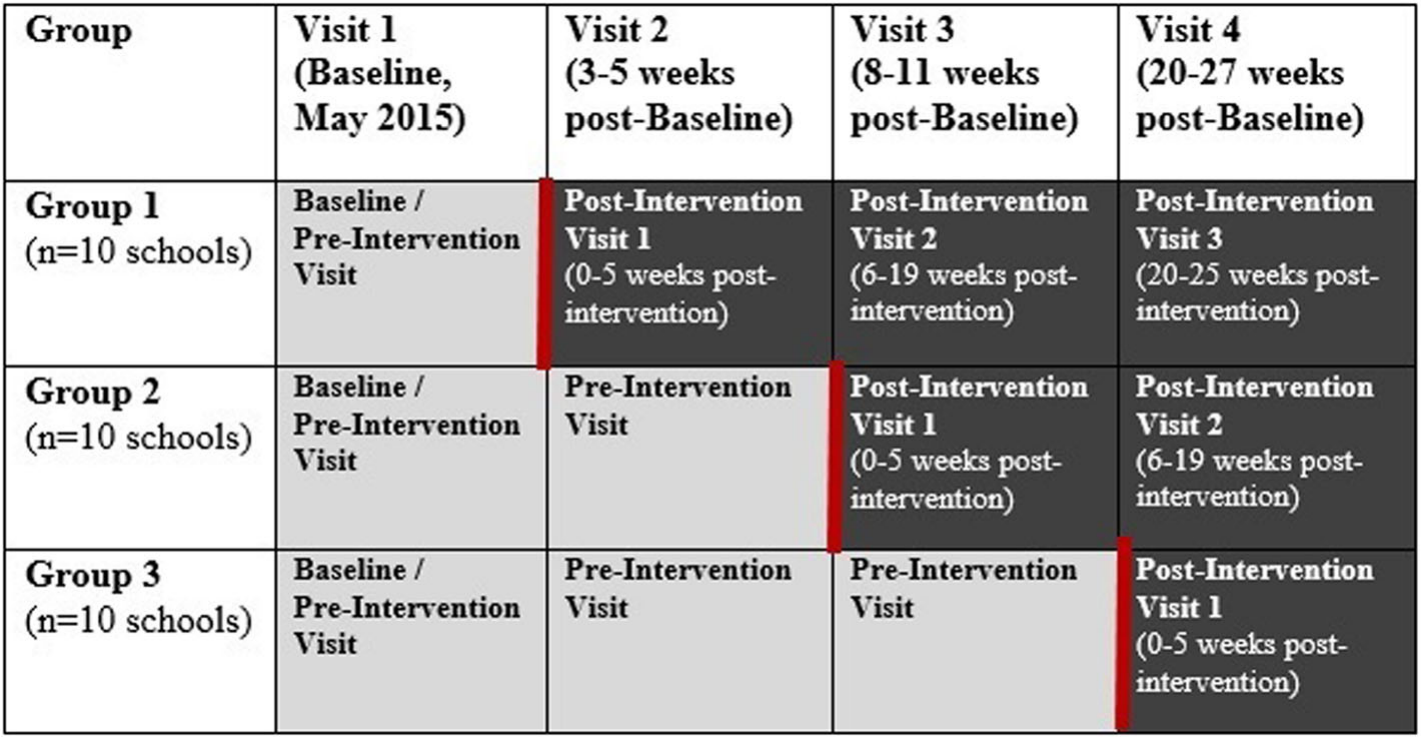
Timeline of the Povu Poa Handwashing Stations - School Pilot Study. Legend: Pre-intervention (“Control group”) data collection.  Intervention delivery.  Post-intervention (“Intervention group”) data collection

Consistent with the stepped-wedge design, we first collected baseline data at all schools, then randomly allocated the schools into 3 groups of 10, and rolled out the intervention sequentially by group. The order in which each group of schools received the intervention was randomly assigned [25]. All data collection and intervention occurred between May and November 2015 (Fig. 1).

### Development of the intervention, pilot tests and training the enumerators

We approached the intervention based on existing theories of behavior change [26]. We hypothesized that there were physical and psychosocial factors that influenced handwashing behavior, and that a handwashing promotion intervention had the ability to change these physical and psychosocial factors, leading to the adoption of target handwashing behaviors. Each study may have a set of setting-specific physical and psychosocial factors for handwashing, which can be idenfied in the early stage of intervention development.

**Fig. 2:**
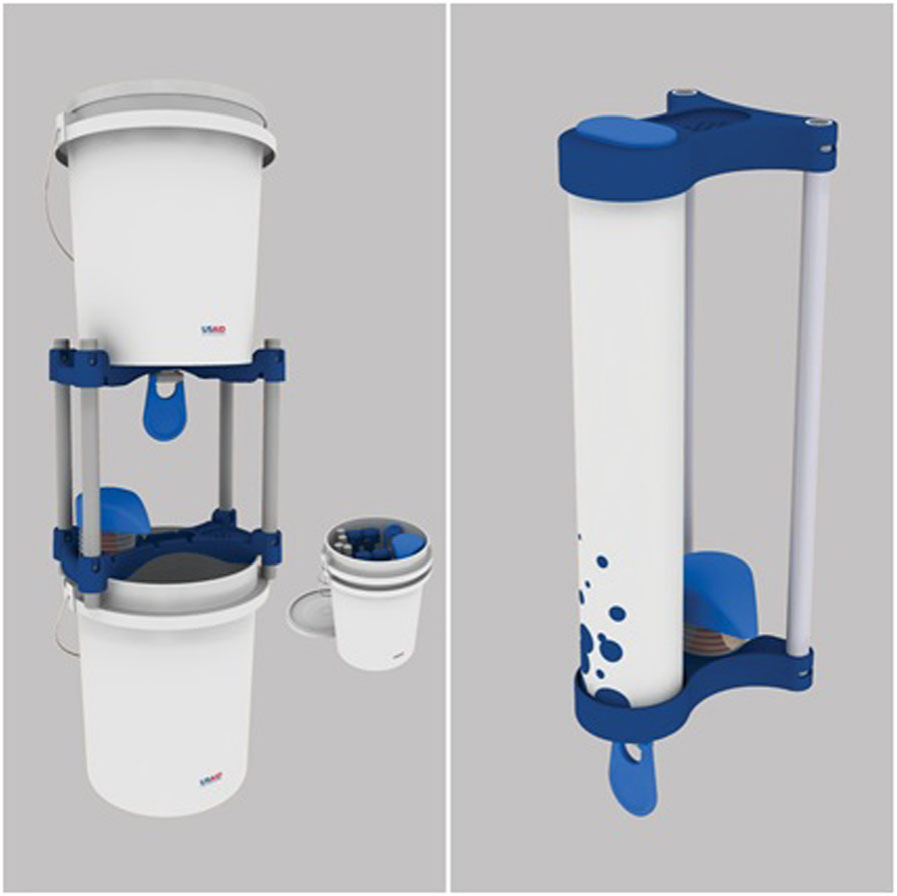
Povu Poa Handwashing System (left: Bucket Model Design; right: Pipe Model Design, reproduced from Whinnery et al., 2016)

We conducted in-depth interviews and focus-group discussions to elucidate factors influencing handwashing behavior with household members, students, and teachers in the study area. The non-profit Catapult Design developed the soapy water handwashing station (SW-HWS) and pilot-tested the preliminary designs in the study region (see Whinnery et al. [18] for additional details on the design process). After multiple iterations, we finalized two designs under the brand Povu Poa (“Cool Foam” in Swahili). The first design was the “Povu Poa bucket model” and the second was the “Povu Poa pipe model”. Both models dispense water and foaming soap. The bucket model serves as an independent structure and holds 20 l of water while the pipe model must be hung from a standing structure, holds up to 5 l of water, and can be plumbed to water tanks and a drainage system (Fig. 2) [18].

To motivate handwashing behavior in schools, we designed an intervention based on disgust and social norms, both of which are strong drivers of handwashing behaviors [24, 27]. We developed an intervention design based on an approach shown to be effective among mothers in rural communities in India [24] and triggering activities designed by UNICEF for handwashing promotion in Malawi [28]. Neither of these interventions had been previously tested among schoolchildren.

We integrated schoolchildren and community members into the intervention development process. First, we pilot-tested the Povu Poa device in 3 schools to determine the feasibility of using the device for handwashing in school setting. We pilot-tested the behavioral intervention in 2 of the 3 Povu Poa pilot schools and used feedback from students to develop the second version. We then presented the second version of the behavioral intervention to local community members and conducted focus group discussions to assess reception and cultural suitability and developed the final version, which consisted of three parts. The details of each part of the intervention are described in Table 1.

**Table 1:** Details of the three-part handwashing behavior change intervention, the Povu Poa Handwashing Stations School Pilot Study

Part	Aim	Details
Part 1: The “Toilet and Shake” Skit	To induce and use disgust as an emotional driver for handwashing	• The resource person pretended to go to the toilet and came back to the assembly without washing hands - simply wiping hands with toilet papers
		• The resource person attempted to shake hands with the students
		• The resource person asked questions about having dirty hands
Part 2: The Handwashing Song	To remind the students to wash their hands every time after using the latrine and before eating	• The resource person taught the students a song about handwashing with soap after using the latrine and before eating
Part 3: The Pledge	To promote a social norm of handwashing	• The resource person asked students to raise hands and say an oral pledge to always wash their hands every time after using the toilet
		• The purpose of the pledging process was to create a social norm (specifically, injunctive norm) for handwashing - to make the students expect others to wash their hands and be aware that others also expect them to wash their hands, creating mutual expectation for handwashing after toilet use
		• The pledge was meant to change the students’ social norms which could then drive their handwashing behaviors

### Sampling frame

We obtained a list of public primary schools in Kisumu County from the local Kenyan Ministry of Education office and used the list as the sampling frame. Thirty (*n* = 30) schools in Kisumu County were randomly selected using a computerized random number generator.

### Eligibility criteria

To meet the eligibility criteria, the school must 1) be public; 2) be a day school, without boarding students; 3) have an existing handwashing system with soap and water; 4) have at least 200 students, and 5) lack access to a piped connection providing continuous water supply (schools with intermittent water supply were eligible).

### Enumerator training

We recruited enumerators from Innovations for Poverty Action - Kenya’s roster of local staff. Enumerator training lasted approximately 1 week and included overview of the study, codes of conduct at the target schools, detailed training on intervention delivery, detailed training on the study instrument, and protocols for data collection and quality control.

### Baseline data collection

Trained enumerators approached the randomly selected schools, asked to meet with the head teacher to explain the study and sought consent for the school to enroll in the study. The consent form included information on purpose of the study, intervention delivery, interviews and observations to be made at the school, follow-up visits, benefits and risk from the study, confidentiality, voluntary nature of participation, and contact details of research staff. The Maseno Universty Ethics Review Committee approved of the procedures for this study. MUERC No.: MSU/DRPC/MUERC/000099/14.

After obtaining consent, the enumerators collected baseline data by interviewing the head teachers and the teachers responsible for school water, sanitation and hygiene. The content of the interview included characteristics of the school, availability of water and soap, availability of budget for soap purchase, and activities related to water, sanitation and hygiene at the school. Schools that met the eligibility criteria were included in the study. One sampled school was ineligible because it had fewer than 200 students, and we replaced the school with another randomly selected school.

In addition to the interviews, the enumerators also conducted rapid observation of handwashing place, where the enumerators observed the presence of soap and water at the school’s handwashing place. After observation of handwashing place, the enumerators also conducted structured observation of handwashing behavior among schoolchildren. We trained the enumerator to position themselves in a discreet location that was adequately far away from the handwashing palce to not be immediately noticed, but still had a direct line of sight to the handwashing place. Enumerators then followed the instructions in the data collection instrument, and observed handwashing behavior after toilet use among students for 1 h or until there were 10 observed events, whichever came first. These observations of handwashing place for presence of soap and water and observations of handwashing behavior after toilet use were repeated during each of the additional visits to the school.

### Intervention delivery

We delivered the intervention in sequence: to the first group of 10 randomly-selected schools (Group 1) in early-June 2015, the second group of 10 schools (Group 2) in early-July 2015, and the third group of 10 schools (Group 3) in mid-October 2015. Group 1 received the intervention 3–5 weeks after the baseline period. Group 2 received the intervention 6–8 weeks after baseline. Group 3 received the intervention 19–24 weeks after baseline.

On the day that the intervention was implemented, enumerators provided the teacher and student leaders responsible for water, sanitation and hygiene activities with 2 soapy water handwashing stations and briefed them on use and maintenance. Enumerators also delivered the handwashing behavioral intervention to the entire school via school-wide assembly (Table 1). The behavior change intervention took between 30 to 60 min to deliver. We implemented all three parts of the behavior change intervention together on the same day with no repetition. The entire intervention delivery required approximately 2 h and could be delivered to an entire group of 10 schools in 4 to 7 business days. All schools in Group 1 (10 schools) and half of all schools in Group 3 (5 schools) received the Bucket Model, while the other 15 schools (all of Group 2 and half of Group 3) received the Pipe Model. Placement of the handwashing station was subject to the discretion of the school staff.

### Follow-up data collection

Study team members made 3 rounds of follow-up visits to each participating school after baseline (Fig. 1). The availability of any type of soap or soapy water (hereafter referred to as “soap”) and water at the handwashing place, and observed handwashing behaviors among schoolchildren were measured in all schools, either at the Povu Poa handwashing station, the toilet or feeding area (intervention schools) or near available water points or handwashing stations (schools not yet receiving intervention). Study team members monitored the condition of Povu Poa Handwashing Station equipment using rapid observation form containing questions on conditions of the handwashing station and conditions of the soap foamer dispenser and replaced or fixed parts that were damaged, cracked or lost.

### Statistical analyses

We used descriptive statistics and log-binomial regression analyses to assess the effect of the intervention on two outcomes: 1) availability of soap and water at the school’s handwashing place; and 2) handwashing with water (and soap) after toilet use during structured observation. We used descriptive statistics to compare outcomes in participating schools after receiving the intervention (post-intervention schools) to outcomes in the same participating schools before receiving the intervention (pre-intervention schools). We then used log-binomial regression to calculate prevalence ratios (PR) and 95% confidence interval. We used the school ID number as the repeated variable to account for the effect of clustering at the school level. We stratified the analyses by levels of co-variables to assess for effect modification. All analyses were conducted using SAS version 9.4.

## Results

The characteristics of the participating schools are summarized in Table 2. Every school had at least one source of water for handwashing, with rainwater storage tank being most common. Of 30 participating schools, 8 had a budget to purchase handwashing soap or hand sanitizer, 17 had received an intervention program promoting water, sanitation and hygiene (WASH) within the past five years. Nearly all (28 schools) had a WASH club, and in 19 schools the WASH club promoted messages about handwashing.

**Table 2:** Characteristics of the participating schools at baseline (*n* = 30 schools)

Item	Baseline
Total number of students at the school (median, IQR)	476 (400, 554)
The school was connected to electrical power lines	23 (76.7%)
Source of water for handwashing	
Rainwater with tank	26 (86.7%)
Groundwater	17 (56.7%)
Students being water from home (carry water in a jerry can)	3 (10.0%)
Water pipelines	2 (6.7%)
The school had no water for at least 30 min at least once/week	
During Term 1: January–April	12 (40.0%)
During Term 2: May–August	7 (23.3%)
During Term 3: September–November	10 (33.3%)
The school had budget to purchase soap or sanitizer?(% yes)	8 (26.7%)
WaSH-related Programs Implemented in Schools	
Received WASH-related program within past five years	17 (56.7%)
On-going WASH-related program	5 (16.7%)
Received hand hygiene-related program within past five years	11 (36.7%)
Interview with WASH club teacher (n = 29)^a^	
School has a WASH club	28 (93.3%)
WASH club organized at least one activity during previous term	21 (72.4%)
WASH club promoted handwashing during previous term	19 (65.5%)
WASH club posted handwashing promotion material	2 (6.9%)
WASH club organized other WASH-related activities	17 (58.6%)
If yes, please describe	
Maintenance of water point and toilets	5 (17.2%)
Promotion of personal hygiene	8 (27.6%)

^a^An interview could not be conducted in school coded “S28”: teacher in charge of WASH activity refused to participate

**Fig. 3:**
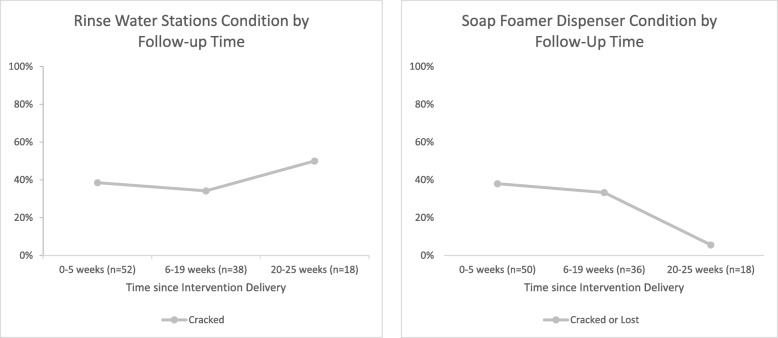
Visible cracking or malfunction of soapy-water handwashing station (at least one component) after the delivery of soapy-water handwashing station and behavior change intervention in public primary schools in Kisumu County, Kenya

Post-intervention schools had significantly higher probability of having water and soap at the handwashing place compared to pre-intervention schools (Table 3). However, by the time of the first post-intervention follow-up visit, 39% of rinse water stations and 38% of soap foam dispensers had at least one part that was cracked (Fig. 3). Answers from the semi-structured qualitative interviews at the final follow-up visit suggested that the top bucket could be heavy for students to fill with water and lift onto the frame, and students did not consistently make the soapy water and re-fill the container when empty.

**Table 3:** Prevalence and PR (95% CI) of having water and soap at handwashing place at 30 participating public primary schools in Kisumu County, Kenya, by pre-intervention vs. post-intervention status

Item	Pre-intervention visits	Post-intervention visits			
		Post-Intervention, all visits	Post-Intervention Visit 1 (0–5 weeks after intervention)	Post-Intervention Visit 2 (6–19 weeks after intervention)	Post-Intervention Visit 3 (20–25 weeks after intervention)
*All schools*					
*Availability of soap and water at the school*	(*n* = 60 school visits)	(*n* = 60 school visits)	(*n* = 30 school visits)	(*n* = 20 school visits)	(*n* = 10 school visits)
Proportion of school visits during which soap and water was observed at ≥1 handwashing place	1 (1.7%)	25 (41.7%)	17 (56.7%)	4 (25.0%)	4 (40.0%)
*Availability of soap and water at the handwashing place* ^a^	(*n* = 139 observations in 60 school visits)	(*n* = 117 observations in 60 school visits)	(*n* = 58 observations in 29 school visits)	(*n* = 40 observations in 20 school visits)	(*n* = 19 observations in 10 school visits)
None	28 (20.1%)	13 (11.0%)	9 (15.5%)	4 (10.0%)	0 (0.0%)
Water only	89 (64.0%)	22 (18.6%)	9 (15.5%)	13 (32.5%)	0 (0.0%)
Soap only	0 (0.0%)	6 (5.1%)	2 (3.4%)	3 (7.5%)	1 (5.0%)
Both soap and water	1 (0.7%)	41 (34.7%)	29 (50.0%)	5 (12.5%)	7 (35.0%)
Don’t know / Couldn’t observe	21 (15.1%)	36 (30.5%)	9 (15.5%)	15 (37.5%)	12 (60.0%)
*PR for having water and soap at handwashing place (*vs. other status)*					
Having water and soap at HW place^a^	*Ref.*	117.00 (16.08, 851.30)	169.65 (21.34, 1348.55)	29.25 (3.21, 266.61)	819.00 (44.4015108.02)

^a^Remark: Excluded “Couldn’t observe” and “Don’t know”, accounted for clustering by school ID

Structured observation included observation of 461 toilet use events in pre-intervention schools, and 383 toilet use events in post-intervention schools. There was a similar proportion of female and male students at both pre-intervention and post-intervention schools, and a similar proportion of younger students (age ≤ 10 years) and older students (age > 10 years) (Table 4). In pre-intervention schools, handwashing with water was observed in only 11% of toilet use events, and there was no handwashing with soap. In post-intervention schools, any type of handwashing (handwashing with water alone or with soap and water) was observed in 62% of toilet use events (PR = 5.96; 95% CI = 3.02, 11.76)), and handwashing with soap was observed in 26% of toilet use events (Table 5). The pre-intervention vs. post-intervention differences in probability of any type of handwashing were slightly greater among children aged ≤10 years (5% vs. 65%; PR = 15.95; 95% CI = 5.57, 45.65) than among children aged > 10 years (18% vs 56%, PR = 3.49; 95% CI = 1.20, 6.34) (Table 5). When there was water and soap at ≥1 handwashing place during rapid observation, the pre-intervention vs. post-intervention differences in probability of observed handwashing was approximately two-folds (42% vs. 76%; PR = 1.98; 95% CI = 1.74, 2.25). When there was no water and soap at any handwashing place during rapid observation, the pre-intervention vs. post-intervention differences in probability of handwashing was approximately six-folds (10% vs. 62%; PR = 6.27; 95% CI = 2.83, 13.91) (Additional file 1: Table S1).

**Table 4:** Observed handwashing behaviors after toilet use among students at participating schools (*n* = 844 toilet use events in 30 schools)

	Pre-intervention (*n* = 461 events in 30 schools)	Post-Intervention Visits			
		Post-intervention overall (*n* = 383 events in 30 schools)	Visit 1 (0–5 weeks) (*n* = 198 events in *n* = 30 schools)	Visit 2 (6–19 weeks) (*n* = 105 events in *n* = 20 schools)	Visit 3 (20–25 weeks) (*n* = 80 events in *n* = 10 schools)
*Age*					
≤ 10 years old	217 (47.1%)	225 (58.7%)	109 (55.1%)	64 (61.0%)	52 (65.0%)
> 10 years old	244 (52.9%)	158 (41.3%)	89 (44.9%)	41 (39.0%)	28 (35.0%)
*Sex*					
Female	231 (50.1%)	203 (53.0%)	105 (53.0%)	54 (51.4%)	44 (55.0%)
Male	230 (49.9%)	180 (47.0%)	93 (47.0%)	51 (48.6%)	36 (45.0%)
*If hands were washed, indicate vigor of scrubbing*	(*n* = 53 events)	(*n* = 237 events)	(*n* = 115 events)	(*n* = 69 events)	(*n* = 52 events)
Vigorously	11 (20.8%)	1 (0.4%)	1 (0.9%)	0 (0.0%)	0 (0.0%)
Moderately	38 (71.7%)	221 (93.2%)	108 (93.9%)	63 (91.3%)	49 (94.2%)
Rinsed/Minimal	4 (7.5%)	15 (6.3%)	6 (5.2%)	6 (8.7%)	3 (5.8%)
*If hands were washed, indicate duration of scrubbing*	(n = 53 events)	(n = 237 events)	(n = 115 events)	(n = 69 events)	(n = 52 events)
Less than 20 s	53 (100.0%)	231 (97.5%)	109 (94.8%)	69 (100.0%)	52 (100.0%)
20 s or more	0 (0.0%)	6 (2.5%)	6 (5.2%)	0 (0.0%)	0 (0.0%)
*If hands were washed, how were hands dried?*	(n = 53 events)	(n = 237 events)	(*n* = 116 events)	(n = 69 events)	(n = 52 events)
Air drying	49 (92.5%)	226 (95.4%)	106 (91.4%)	69 (100%)	51 (98.1%)
Cloth towel	0 (0.0%)	1 (0.4%)	1 (0.9%)	0 (0.0%)	0 (0.0%)
Own clothes	3 (5.7%)	7 (3.0%)	6 (5.2%)	0 (0.0%)	1 (1.9%)
Could not observe	1 (1.9%)	3 (1.3%)	3 (2.6%)	0 (0.0%)	0 (0.0%)

**Table 5:** Observed handwashing behaviors at toileting events by age group of the students (n = 844 toilet use events in 30 schools)

	Pre-intervention	Post-Intervention Visits			
		Post-intervention overall	Visit 1 (0–5 weeks since intervention)	Visit 2 (6–19 weeks since intervention)	Visit 3 (20–25 weeks since intervention)
*Observed handwashing behavior, all events*	(n = 461 events in 30 schools)	(n = 383 events in 30 schools)	(n = 198 events in n = 30 schools)	(n = 105 events in n = 20 schools)	(n = 80 events in n = 10 schools)
No handwashing	354 (76.7%)	68 (17.8%)	51 (25.8%)	17 (16.2%)	0 (0%)
Handwashing with water only	53 (11.4%)	138 (36.0%)	60 (30.3%)	46 (43.8%)	32 (40%)
Handwashing with water and soap	0 (0.0%)	98 (25.6%)	55 (27.8%)	23 (21.9%)	20 (25%)
Could not observe	54 (11.7%)	79 (20.6%)	32 (16.2%)	19 (18.1%)	28 (35%)
*PR for observed handwashing (*vs. *no handwashing), all events*					
Observed handwashing^a^	*Ref.*	5.96 (3.02, 11.76)	5.32 (2.61, 10.83)	6.16 (2.96, 12.82)	N/A**
*Observed handwashing behavior,* age ≤ 10 years old (*n* = 442 events)	*(n = 217 events)*	*(n = 225 events)*	*(n = 109 events)*	*(n = 64 events)*	*(n = 52 events)*
No handwashing	181 (83.4%)	29 (12.9%)	19 (17.4%)	10 (15.6%)	0 (0.0%)
Handwashing with water only	10 (4.6%)	86 (38.2%)	33 (30.3%)	29 (45.3%)	24 (46.2%)
Handwashing with water and soap	0 (0.0%)	61 (27.1%)	36 (33.0%)	12 (18.8%)	13 (25.0%)
Could not observe	26 (12.0%)	49 (21.8%)	21 (19.3%)	13 (20.3%)	15 (28.8%)
*PR for observed handwashing (*vs. *no handwashing),* age ≤ 10 years old					
Observed handwashing^a^	*Ref.*	15.95 (5.57, 45.65)	14.98 (5.11, 43.90)	15.35 (5.20, 45.30)	N/A^b^
*Observed handwashing behavior,* age > 10 years old (*n* = 402 events)	*(n = 244 events)*	*(n = 158 events)*	*(n = 89 events)*	*(n = 41 events)*	*(n = 28 events)*
No handwashing	173 (70.9%)	39 (24.7%)	32 (36.0%)	7 (17.1%)	0 (0.0%)
Handwashing with water only	43 (17.6%)	52 (32.9%)	27 (30.3%)	17 (41.5%)	8 (28.6%)
Handwashing with water and soap	0 (0.0%)	37 (23.4%)	19 (21.3%)	11 (26.8%)	7 (25.0%)
Could not observe	28 (11.5%)	30 (19.0%)	11 (12.4%)	6 (14.6%)	13 (46.4%)
*PR for observed handwashing (*vs. *no handwashing),* age > 10 years old					
Observed handwashing^a^	*Ref.*	3.49 (1.2, 6.34)	2.96 (1.57, 5.59)	4.02 (2.03, 7.94)	N/A^b^

^a^Accounted for clustering by school, hands washed with either water only or with water and soap

^b^RR (95% CI) for the outcome not available due to perfect prediction

## Discussion

Our study assessed the effectiveness of two major innovations to motivate handwashing: a cost-saving soap foam dispenser with a water-frugal tap, and a behavior change intervention aimed at triggering disgust and promoting handwashing as a social norm. This study is the first to assess the effect of both interventions delivered in combination at schools in a low-income country setting. Availability of water and soap at handwashing place and observed handwashing after toilet use were significantly higher after receiving the intervention than before the intervention. Observed handwashing with water and soap increased from non-existent to 26% of toilet use events. In the study area, WASH (water, sanitation and hygiene) clubs are clubs of students with one supervising teacher each with the purpose to engage schoolchildren in activities related to water, sanitation and hygiene. Schools that had an active WASH club might have a more rapid adaptation of the intervention than schools that did not have a WASH club because the club’s purpose aligned with implementation of the intervention.

The behavior change intervention activities in our study aimed to address multiple drivers of behavior, including injunctive norm, i.e., the perception of what is commonly approved and disapproved among relevant others [29]. The “Toilet and Shake” Skit in Part 1 of the intervention aimed to induce and use disgust as an emotional driver for handwashing. However, the skit itself could lead to a change in: a) Disgust and propensity and/or disgust sensitivity [30]; b) Perceived susceptibility to diarrheal disease; c) Injuctive or descriptive social norms, and/or; d) Self-efficacy to refuse to shake hands with someone coming out of the toilet.

With regards to disgust and social norms, after the “Toilet and Shake” Skit, there was a session to teach handwashing song and a handwashing pledge. The aim of the song and the public pledge was to create a sense of awareness that each student’s peers also had disgust for unclean hands and a commitment for handwashing, which had the potential to lead to formation of injunctive norm in each participating student. Experimental data from a previous study suggested that the use of strong injunctive normative elements as focal point of a behavioral decision can be effective in creating desirable conduct [31]. However, the use of behavior change interventions that target disgust and social norms in the behavior change intervention may have moral and ethical implications, as interventions where disgust is combined with fear can create repulsion of individuals or groups who are positioned as disgusting [32]. In addition, the content of the pledge itself could induce a sense of commitment on the part of the participating student [33]. Commitment comes from internal pressure to engage in a behavior, so although the intervention was delivered in large group, the pressure to wash hands with soap could be both external and internal.

Our study only measured handwashing-related outcomes and did not measure changes in any of the potential psychosocial determinants of handwashing. We do not know which psychosocial behavioral determinants, or a combination thereof, were affected by the intervention and were associated with changes in handwashing behavior. Limitations with regards to lack of measurement of psychosocial drivers of handwashing behavior and negative consequences of the interventions should be taken into consideration when assessing suitability of this type of intervention.

Post-intervention availability of handwashing materials was higher in this study than in the 2012 evaluation of a school and household handwashing promotion program implemented in the same region of Kenya, which used health education based on a soap mascot, and found that only 13% of schools had a handwashing place with soap and water at post-intervention [11]. The use of the foaming soap dispenser and water-frugal tap incurs a lower cost of handwashing consumable materials than conventional water and bar soap handwashing stations in Kenya ($0.10 USD per 100 uses vs. $0.20 to $0.44 USD per 100 uses, respectively) [18]. With an estimated sale price of <$20 USD, the savings from reduced quantities of soap and water needed for handwashing with the Povu Poa could cover its own capital cost in just a few months in a school, facilitating scale up [18]. Furthermore, the Povu Poa securely stores soapy water for conversion into foam, which helps to deter soap theft or loss, which are common in school hand hygiene [16, 19]. The results of our study supported the findings in previous studies that soapy water can be an affordable alternative to bar soap [19, 34].

However, at post-intervention, approximately half of all observed handwashing systems still did not have water and soapy water available for handwashing. Anecdotally, field staff reported that the caps and water taps were not resistant to ultraviolet radiation from the sun and became brittle and cracked, which may have affected functionality. This cracking might have been prevented by using UV resistant plastic to manufacture the soap dispenser caps and water dispenser taps. There were also more than 400 students in three-fourths of the participating schools, thus the student-per-handwashing-station ratio was at least 200:1 contributing to crowding and frequent usage. A general guideline for the student-per-handwashing-station ratio does not exist, but the guideline for sanitation recommends 20–40 students per latrine [35]. Providing two stations per school was possibly insufficient, and providing more stations may be needed to manage crowding and reduce wear and tear on the product.

Probability of handwashing with soap after toileting at the first, second and third post-intervention visits were all significantly higher than the probability of handwashing with soap pre-intervention, suggesting that the effect could last beyond the study period. In that regard, stepped-wedge trials are not free from potential biases, particularly when there are secular trends associated with the study outcome [36]. However, these potential biases were unlikely to affect the validity of the study findings, given the extent of post-intervention vs. pre-intervention differences. Improvement in handwashing behavior was greater among children ≤10 years old than among children > 10 years old, consistent with findings from an urban US setting [37]. Children in the younger age group might have been more prone to change their behavior than those in the older age group. Considering that primary public schools in Kenya include students from kindergarten to Year 8 (age 4 thru 14) and the rapidly growing population, the proposed intervention is relevant to the study setting.

Even at post-intervention, only one-third (32%) of observed toilet use events was followed by handwashing with soap. Although the change in probability of handwashing with soap after toilet use was significant, it still was less than ideal. As mentioned earlier, the theoretical framework for the intervention posits that the intervention changes physical and psychosocial factors for handwashing, which then drives compliance to handwashing after toilet use. The data showed that the physical factors, i.e., the availability of water and soap for handwashing, improved after the intervention. According to the theoretical framework, it is possible that the intervention might not have been ideal in changing psychosocial factors, or the psychosocial factors that were targeted by the intervention were not strong determinants of handwashing in our study setting.

Future studies should consider other interventions that yield high probability of handwashing with soap at post-intervention. A 6-week pilot intervention study in Bangladesh that employed only visual cues (nudges) increased the probability of handwashing with soap from 4% at baseline to 68% post-intervention [38]. Incorporating visual cues (nudges) into the intervention could yield larger gains in handwashing behavior. In addition, our study results showed that handwashing still happened in post-intervention schools even when water and soap were not available at the handwashing place during rapid observation. Possible explanations included that the students could have made soapy water and filled the soap foamer after the rapid observation, or the students could have brought soap from elsewhere to the handwashing place. Future intervention studies should also consider the components of intervention fidelity to put the findings into context: 1) whether the change agents follow the established protocol when delivering the intervention (i.e., adherence); 2) whether the change agents are able to operationalize the behavioral drivers in the intervention to a sufficient degree as to affect behavior change (i.e., quality of delivery); 3) whether the components of the intervention were delivered to the intended number of target population (dosage and exposure); 4) whether the intended response occurs among the target population [39, 40].

There are five major limitations in our study. First, it was not possible to blind the enumerator from the intervention status of the school because our study involved installation of handwashing equipment and the soapy water handwashing stations were the focus location of the structured observation. The enumerators could have introduced observer bias into the study. However, we trained the enumerators to adhere strictly to established observation protocols and there was no incentive for the enumerator to over-report handwashing behavior in the post-intervention period. Second, we could not observe handwashing behavior following 12% of toileting events in pre-intervention schools and 21% of toileting events in post-intervention schools (up to 29% in the final visit in schools that received three post-intervention follow-up visits) because there were multiple children or events to observe at the same time, and the children might crowd around the handwashing station, making detailed observation difficult. These non-observations were classified as missing data. If the non-observations did not occur at random, then it could have biased our estimate of the effect of the intervention either away from the null or toward the null, depending on what was not observed during the pre-intervention period and the post-intervention period. However, non-observation is unlikely to be non-random because the interviewers were trained to follow established observation procedure that was identical for pre-intervention and post-intervention schools. Third, our study did not include measurement of psychosocial drivers (disgust, social norms, commitment, perceived susceptibility to diarrheal diseases, self-efficacy, or other psychosocial determinants of handwashing). We could not assess the effect of the intervention according to the theory of change, thus we could not conclude whether the observed effect of the intervention happened through changes in disgust, social norms, other psychosocial drivers of handwashing, or a combination thereof.Fourth, our observation of handwashing behaviors did not include non-toileting events, such as handwashing before eating and after touching respiratory fluids. Therefore, we cannot comment on the efficacy of the intervention for handwashing behavior change at other times of possible pathogen transmission. Lastly, we conducted this study in public primary schools in one county in the western part of Kenya, and thus, the results may not be generalizable to other settings with different infrastructural and social contexts. Future studies should consider: 1) interventions that can further improve handwashing behavior, aiming for > 90% handwashing compliance after toileting; 2) replication of the study to include all pathogen hand transmission events on a larger scale in other low-income country settings, and; 3) measurement of pre-intervention and post-intervention social norms if the intervention includes a component that aims to change social norms.

## Conclusions

A stepped wedge cluster randomized trial at public primary schools in western Kenya had an intervention that included providing water-frugal handwashing system with foaming soap and a behavior change program employing disgust, norms, and pledging. Comparing pre-intervention outcomes to post-intervention outcomes, the intervention improved availability of soap and water at handwashing places, and enhanced the probability of handwashing with water and soap after toilet use among schoolchildren.

## Additional file

Additional file 1: **Table S1.** Observed handwashing behaviors at toileting events according to whether there was at least one handwashing place with water and soap during rapid observation (*n* = 844 toilet use events in 30 schools). (DOCX 35 kb)

## Abbreviations

CI: Confidence interval
MUERC: Maseno University Ethics Review Committee
PR: Prevalence ratio
SW-HWS: Soapy water handwashing station
UNICEF: United Nations Children’s Fund
USD: United States dollar
UV: Ultraviolet radiation
WASH: Water, sanitation and hygiene
