# Supplementary material for: Effect of an equipment-behavior change intervention on handwashing behavior among primary school children in Kenya: the Povu Poa school pilot study
Source: BMC Public Health. 2019 May 28;19:647. doi: 10.1186/s12889-019-6902-2 (PMC6537192; doi:10.1186/s12889-019-6902-2)
Supplement: Supplementary file 1 — Table S1. Observed handwashing behaviors at toileting events according to whether there was at least one handwashing place with water and soap during rapid observation (n = 844 toilet use events in 30 schools). (DOCX 35 kb) [file 12889_2019_6902_MOESM1_ESM.docx]

# **Supplementary Table 1**. Observed handwashing behaviors at toileting events according to whether there was at least one handwashing place with water and soap during rapid observation (n=844 toilet use events in 30 schools)

|  | **Pre-intervention** |  | **Post-Intervention Visits** | | | | |
| --- | --- | --- | --- | --- | --- | --- | --- |
|  |  |  | **Post-intervention overall** |  | **Visit 1**  **(0-5 weeks)** | **Visit 2**  **(6-19 weeks)** | **Visit 3**  **(20-25 weeks)** |
| ***Observed handwashing behavior when there was water and soap at ≥ 1 handwashing place*** | *(n=19 events)* |  | *(n=153 events)* |  | *(n=96 events)* | *(n=24 events)* | *(n=33 events)* |
| No handwashing | 11 (57.9%) |  | 23 (15.0%) |  | 23 (24.0%) | 0 (0.0%) | 0 (0.0%) |
| Handwashing with water only | 8 (42.1%) |  | 39 (25.5%) |  | 16 (16.7%) | 7 (29.2%) | 16 (48.5%) |
| Handwashing with water and soap | 0 (0.0%) |  | 77 (50.3%) |  | 48 (50.0%) | 15 (62.5%) | 14 (42.4%) |
| Could not observe | 0 (0.0%) |  | 14 (9.2%) |  | 9 (9.4%) | 2 (8.3%) | 3 (9.1%) |
| *PR for observed handwashing (vs. no handwashing)* |  |  |  |  |  |  |  |
| Observed handwashing* | *Ref.* |  | 1.98 (1.74, 2.25) |  | 1.75 (1.41, 2.17) | N/A** | N/A** |
| ***Observed handwashing behavior when there was no water and soap at any handwashing place*** | *(n=442 events)* |  | *(n=230 events)* |  | *(n=102 events)* | *(n=81 events)* | *(n=47 events)* |
| No handwashing | 343 (77.6%) |  | 45 (19.6%) |  | 28 (27.5%) | 17 (21.0%) | 0 (0.0%) |
| Handwashing with water only | 45 (10.2%) |  | 99 (43.0%) |  | 44 (43.1%) | 39 (48.1%) | 16 (34.0%) |
| Handwashing with water and soap | 0 (0.0%) |  | 21 (9.1%) |  | 7 (6.9%) | 8 (9.9%) | 6 (12.8%) |
| Could not observe | 54 (12.2%) |  | 65 (28.3%) |  | 23 (22.5%) | 17 (21.0%) | 25 (53.2%) |
| *PR for observed handwashing (vs. no handwashing)* |  |  |  |  |  |  |  |
| Observed handwashing* | *Ref.* |  | 6.27 (2.83, 13.91) |  | 5.57 (2.28, 13.61) | 6.33 (2.66, 15.05) | N/A** |

*Accounted for clustering by school, hands washed with either water only or with water and soap

**RR (95% CI) for the outcome not available due to perfect prediction
